# Increasing phosphorus rate alters microbial dynamics and soil available P in a Lixisol of Zimbabwe

**DOI:** 10.1371/journal.pone.0291226

**Published:** 2023-09-08

**Authors:** Tonny P. Tauro, Hatirarami Nezomba, Florence Mtambanengwe, Paul Mapfumo

**Affiliations:** 1 Department of Soil Science & Environment, University of Zimbabwe, Harare, Zimbabwe; 2 Department of Natural Resources Management, Marondera University of Agricultural Sciences & Technology, Marondera, Zimbabwe; Indian Agricultural Research Institute, INDIA

## Abstract

Soil phosphorus (P) deficiency is a major challenge to food security in most parts of sub-Saharan Africa, including Zimbabwe, where farmers largely depend on local organic nutrient resources as fertilizer in the production of crops. Soil microorganisms can contribute to synchronous availability of soil P to plants through regulating immobilization and mineralization cycles of soil P pools but their activity may be influenced by antecedent soil P, P fertilizer application regimes and P uptake by plants. Using soils collected from plots where *Crotalaria juncea* (high quality), *Calliandra calothyrsus* (medium quality), cattle manure (variable quality), maize stover and *Pinus patula* sawdust (both low quality) were applied at the rate of 4 t C ha^-1^ with 16 kg P ha^-1^ at the start of every season over 16 seasons. A pot study was conducted to evaluate the influence of increasing inorganic P fertilizer rates (26 and 36 kg P ha^-1^) on soil microbial dynamics, soil P pools, and maize P uptake. Results indicated that nineteen (19) fungal and forty-two (42) bacterial colonies were identified over the study period. Fungi dominated bacteria on day one, with *Aspergillus niger* showing a 30–98% abundance that depends on organic resource quality. Overall, microbial diversity peaked activity characterized succession on day 29, which coincided with a significant (P<0.05) increase in P availability. Increasing P rate to 26 kg P ha^-1^ amplified the microbial diverse peak activity under medium-high quality resources while under the control the peak emerged earlier on day 15. *Mucor* and *Bacillus* had peak abundances on day 43 and 57, respectively, across treatments regardless of P rates. Treatment and P rate had a significant (P<0.01) effect on microbial P. Bacteria were more responsive to added P than fungi. Increasing P to 36 kg P ha^-1^ also stimulated an earlier microbial diverse peak activity under maize stover on day 15. Addition of P alone, without supplying complementary nutrients such as N, did not have a positive effect on maize P uptake. Farmers need to co-apply medium-high quality organic resources with high fertilizer P rates to increase microbial diversity, plant available P and maize growth on sandy soils (Lixisols). Our results suggest that there is a need to reconsider existing P fertilizer recommendations, currently pegged at between 26 and 30 kg P ha^-1^, for maize production on sandy soils as well as develop new fertilizer formulations to intensify crop production in Zimbabwe.

## Introduction

Most smallholder farmers in sub-Saharan Africa (SSA) are in a vicious food insecurity cycle largely because they grow crops on degraded soils that are inherently low in primary and secondary nutrients to support meaningful food and feed production [1–3]. For example, > 70% of the rural population in Zimbabwe conduct crop production on sandy soils where maize, the staple crop in the country, yield as low as 1t grain ha^-1^ [2, 4]. These low crop yields typify most smallholder farming areas of Southern Africa dominated by sandy soils [5, 6]. Soil acidity is one of the major fertility challenges exhibited by sandy soils. The acidity challenge often leads to poor establishment and growth of both cereal and legume crops [7, 8]. In addition, sandy soils have low soil organic matter arising from low application of organic resources due to a number of reasons [9]. This worsens the acidity problem as organic nutrient resources such as livestock manure and ash are known to reduce soil acidity. The combination of low soil organic carbon (< 0.5%) and acidity limits microbial survival that are key in nutrient cycling /availability and consequently affecting establishment and growth of major crops [10].

Apart from acidity and low soil organic matter, P deficiency affect cereal/ legume establishment and yields [11] thus ranked second, third and fourth most limiting nutrient in East Africa, West Africa and Great Lakes region, respectively [12]. Highly weathered and old soils derived from parent materials with low P reserves (e.g., granites, gneiss, aeolian), have low plant available P in most cropped fields across SSA [13, 14]. Efforts to increase plant available P through addition of organic resources is limited by low P content in most organics, seasonal variation in quality and immobilization, while decomposition is also curtailed by low nitrogen supply [15, 16]. As such, application of high rates of soluble inorganic phosphate fertilizer, as done in Asia, is the major realistic option for increasing plant available P and cereal yields [17]. However, the cost of NPK-based fertilizers in SSA, ranging from $ USD 0.2 to 0.7 kg^-1^, is too high for most farmers. Subsequently, farmers apply less (an average of 5 kg P ha^-1^) than what is removed by crops [17–20] thereby promoting mining of soil P and other nutrients [21]. The peak P theory by Cordell [22] and phosphates production challenges in Zimbabwe cited by Tumbure et al. [23] would further increase prices of P based fertilizers thus compounding the existing soil fertility challenges. Harnessing the ability of soil microbes to solubilize otherwise recalcitrant soil P pools could be a potential option to unlock soil P, and thus ameliorating deficiency. Besides most farmers not being able to apply the recommended P fertilizer rates, an additional constraint to increased crop yields is the unavailability of soil P to plants due to its complexation by various biological, physical and chemical processes in soils [24]. Agronomic options for increasing plant available P, and improving its utilization and recovery are thus key. A number of studies have reported positive correlation between plant available P and crop yields [25–27]. Given the link between P and food and feed security it is essential to find options of increasing soil available P, and improving its utilization and recovery.

Technologies such as sequences of Integrated Soil Fertility Management (ISFM) options, biochar application and co-application of organic and inorganic fertilizers have shown positive impact on nutrient release, building up of plant available P and crop yields in most parts of East and Southern African [28–31]. While direct addition of organic and inorganic fertilizers is key to providing nutrients to plants, macrofauna, fungal and bacteria play an important role in the recycling and recovery of added nutrients e.g. through decomposition, mineralization and immobilization. [32–34]. For example, microbes have been shown to solubilize fixed P in soils as well as increase crop yields [35, 36]. On the other hand, judicious addition of organic and inorganic fertilizers increase soil microbial activity and diversity [33]. However, there is little knowledge on effects of increasing P rates on soil microbial populations and diversity, plant available P and nutrient uptake following long-term ISFM (repeated seasonal application of inorganic and organic fertilizers). In this study we hypothesized that long-term ISFM (> 10 cropping seasons) anchored on seasonal addition of medium-high quality organic nutrient resources and high rates of inorganic P fertilizer significantly increases soil microbial populations and diversity, and plant available P. This study therefore aimed to: (1) determine the influence of increasing mineral P fertilizer rates on microbial dynamics and diversity under ISFM; (2) determine the influence of organic nutrient resources and mineral P fertilizer rates on changes in soil P pools, and maize P uptake under ISFM; and (3) identify factors underpinning changes in microbial community structure.

## Materials and methods

### Study site, biomass generation and experimental design

The study was based on a long-term field experiment established during the 2002/03 season under the NUESOM project ‘*Managing soil organic matter for improved nutrient use efficiency on smallholder farms in Zimbabwe*’ Grant 2002 FS 189, funded by Rockefeller Foundation [37]. In most parts of Zimbabwe and Southern Africa, smallholder farmers utilize organic nutrient resources of different quality applied at varying quantities in combination with inorganic fertilizers to fertilize crops depending on availability. Therefore, this experiment was based on the need to balance crop nutrients demand, availability of nutrient resources (organic and inorganic) and building soil organic matter, while simulating smallholder farmers’ soil fertility management practices. It was also underpinned on repeated co-application of different quality organic resources and inorganic fertilizers with the view of increasing crop N availability and building fertility of degraded Lixisol soils in the short and long term [29, 37]. Lixisols are soils (WRB) in which considerable clay, sesquioxides (iron/aluminium oxides and hydroxides) and colloidal humus have been removed from the A horizon and deposited in subsurfical horizons such as B and C through pedogenetic processes, particularly eluviation [38]. Lixisols are therefore characterized by higher amounts of low-active clays and more exchangeable bases in the subsurfical horizons than the upper horizons. In SSA, Lixisols occupy approximately 220 million ha of land, with more than half of the area under agriculture [38]. The initial physico-chemical properties at establishment of the long-term experiment are shown in S1 Table. *Crotalaria juncea* (hereafter *Crotalaria*), *Calliandra calothyrsus* (hereafter *Calliandra*), cattle manure (hereafter manure), *Zea mays* stover (hereafter maize stover) and *Pinus patula* sawdust (hereafter sawdust) were the five different quality organic resources used in this experiment. In terms of quality, *Crotalaria*, *Calliandra*, manure, maize stover and sawdust represented high, medium, variable, low and very low, respectively (S2 Table), thereby covering the range of organic nutrient resources commonly applied by farmers. According to the organic resources database (ORD) [39], high quality organic materials contain *>* 2.5% N, < 15% g lignin and *<* 4% polyphenols, and have C/N ratio of *<* 30. Medium quality organic materials either have *>* 2.5% N, *>* 15% lignin and *>* 4% g polyphenols, and a C/N ratio of *<* 30, or *<* 2.5%, *<* 15% lignin and *<* 4% polyphenols with a C/N ratio of *>* 30. The low to very low quality organic resources are those with *<* 2.5% N, *>* 15% lignin and *>* 4% polyphenols, and a high C/N ratio of *>* 30. Organic resources were applied at 4.0 t C ha^-1^ in main plots measuring 12 m × 6 m. Incorporation was done to a depth of 0.15–0.20 m using a hoe in early December after the start of the rainy season, which is the normal planting time for most smallholder farmers [40]. An additional main plot without any organic resources applied was added (hereafter referred to as control). Phosphorus, potassium (K) and sulphur (S) were applied to all treatments at 16.0, 14.7 and 4.6 kg ha^-1^, correspondingly, using a basal fertilizer with 14% P: 13.4% K: 5% S [41]. Bulking plus handling of organic nutrient resources and general management of the trial has been the same since 2002/3 season. Contributions of this long-term trial to the body of knowledge on soil macrofauna, soil fertility management and maize productivity trends have been highlighted in several studies [29, 33, 34, 41–44].

### Establishment of pot experiments

Following incorporation of the organic amendments at 4 t C ha^-1^ and broadcasting of basal fertilizers in the 2016/17 season as the long-term trial continued, three composite soil samples (0–0.3m) were collected from six treatments inclusive of the control within each block and placed in 3.5 kg pots. Each treatment had nine (9) pots giving 54 pots with air-dried soil for establishing the experiment at Soil Productivity Research Laboratory (SPRL) in Zimbabwe. SPRL is located approximately 67 km south east of Harare at Grasslands Research Institute in Marondera,(31◦28′56′′E; 18◦10′15′′S). SPRL is a government of Zimbabwe-owned soil microbiology laboratory assigned to manufacture rhizobia inoculants for production of grain legumes and conduct research on other plant growth promoting rhizobacteria (PGPR). As such, no special permission was required for conducting this microbial evaluation study focusing on soil phosphate solubilizing microbes. In addition, the laboratory and surrounding environment are used for agricultural research related to crop production and livestock feed. The climate at SPRL is characterized by mean annual rainfall of between 600 and 900 mm, with mean maximum and minimum temperatures of 23°C and 11°C, respectively.

From the nine (9) pots under each treatment, three (3) pots were randomly selected (using random number tables method) to maintain the 16 kg P ha^-1^. The remaining six (6) pots were randomly selected to establish two (2) treatments with three replicates each by adding 10 kg P ha^-1^ (thereafter 26 kg P ha^-1^) and 20 kg P ha^-1^ (thereafter 36 kg P ha^-1^) using single super phosphate (14% P). The experiment had two factors: three (3) P rates (16, 26 and 36 kg P ha^-1^), five (5) organic nutrient resources (*Crotalaria*, *Calliandra*, manure, maize stover and sawdust) and the control thus making it a 3 x 6 factorial design. Treatments were randomized within each block and replicated three times across three blocks. Three seeds of a maize hybrid cultivar SC555 (approximately 136 days to maturity) were planted in each pot and thinned to two plants per pot one week after crop emergence. To ensure maximum microbial activities, pot moisture was maintained at 70% field capacity with about 500 mL of de-ionized water applied every second day to avoid excess water draining out. Pots were kept weed free by manually pulling out all emerging weeds.

### Soil sampling for microbial and chemical analysis

Following watering the pots, composite soil samples were collected fortnightly starting on day 1, 15, 29, 43 and 57. Such a sampling scheme is capable of capturing the rapid transition of microbes from dormant to active state following substrate addition [45]. To reduce cross contamination, each pot had a specific plastic spatula for collecting soil samples for both chemical and microbial assessment. Seventy percent (70%) alcohol was used to disinfect the plastic spatula and hands during sampling and between sampling of each pot, respectively. Soil samples were stored in a fridge to reduce changes in community structure before analysis [46]. Soil samples were analyzed for available P, microbial P, and pH using Olsen, Chloroform fumigation extraction and 0.01 M CaCl_2_ methods, respectively [47, 48]. Standards were freshly prepared and samples replicated during the routine analyses.

### Estimating population of phosphate solubilizing bacteria and fungi

To estimate fungal and bacteria populations, 0.85% NaCl saline solution was used to isolate soil microbes from a 1 g sub-sample. Following isolation and serial dilutions, culturing was done on Pikovskayas (PVK) agar medium (0.5 g yeast extract; 10 g dextrose; 5 g calcium phosphate; 0.5 g ammonium sulfate; 0.2 g potassium chloride; 0.1 g magnesium sulfate; 0.0001 g manganese sulfate; 0.0001 g ferrous sulfate; 15 g agar; 1 L distilled water; the pH adjusted to 7.0 ± 0.2 before sterilization) [49–51]. The culturing involved utilizing the micropipette and spreader to inoculate the PVK medium in sterile petri dishes using serial dilutions. On day 1, four dilutions (10^−2^, 10^−4^, 10^−6^ and 10^−8^) were made and plated from five randomly selected treatments to establish a working dilution factor for the study. To stimulate microbial growth, the inoculated medium was incubated in sterile incubators at 30° ± 1°C for 5 days or more depending on observations. Colony forming units (CFU) were estimated by enumerating the total colonies within each plate while separating them into fungi and bacteria broad groups according to morphology (size, shape, colour pigment, edge, spreading pattern, opacity and shine, etc.). Plates without colonies were further incubated for 2–4 days while monitoring for colony development before recounting. A dilution factor of 10^−6^ was identified as the most appropriate concentration to separate microbial colonies from the mixed population, but was adjusted depending on observations. Population was calculated per gram on dry soil basis taking into consideration the dilution factor. Some fungal species were identified basing on the cultural properties by Ameh and Kawo [52].

### Determining maize productivity and P uptake

On the last day of soil sampling, maize shoot biomass was determined by cutting the plants at the soil line and oven drying them for 24 hours to constant weight at 65 °C. Maize roots were extracted from the pots by carefully turning moistened soil while removing the roots from the bulky soil. Root dry weight was determined using the oven drying method as was used for the shoots. The dried shoot and root samples were then ground in a Wiley Mill to pass through a 1 mm sieve. To quantify total P in biomass, ground samples were digested using the Micro-Kjeldahl mixture and P colorimetrically measured at wavelength 880 nm [47]. Phosphorus uptake by shoot and root was obtained by multiplying the tissue P concentration by the corresponding biomass.

### Data analyses

Alpha diversity measures i.e., Shannon-Wiener (Hʹ) and evenness (E) [53] were calculated in Paleontological Statistics (PAST) package version 4.02 [54]. Changes in soils attributes (pH, available P, and microbial P) and maize productivity parameters were analyzed using GenStat 22^nd^ Edition [55] with mean comparisons done using Turkey’s test at 95% confidence interval. Multivariate analysis (MVA) techniques were used to establish relationships among microbial composition and environmental factors using CANOCO 4.5 [56]. Data was subjected to gradient analysis and the gradient was 6.1. This meant unimodal pathway and detrended correspondence analysis DCA (CA) were used as appropriate techniques for analysing the data [57]. Environmental factors and quality parameters that aligned with axes had strong effects on species composition. Finally, an interactive-forward test was used to identify the most significant environmental factors explaining the results from DCA analysis.

## Results

### Identified microbes

Across all the five sampling times and treatments, nineteen (19) fungal colonies were identified and seven species classified. Some fungal species transformed morphologically with further incubation time while at the same time both the original and transformed fungi were simultaneously stimulated later during the experiment (Table 1). Forty-two (42) bacteria colony forming units were identified over time across treatments, of which six species produced hallow zones (Table 2). Using simple broad classification, the cream rod shaped bacteria was *Bacillus* while the majority were spherical-shaped (*Cocci*).

**Table 1:** Morphological characteristics of identified fungi from P greenhouse experiment.

Isolates	Morphology	Transformation with time	Species classification	Classified based on
SF_1_	Whitish/light cotton like	Black centre and green edges	*Mucor*	Ameh and Kawo, 2017
SF_2_	White top and pink base			
SF_3_	Milky centre with whitish taints spreading			
SF_4_	Yellowish	Blood red at base and yellow taint spreading,		
SF_5_	Yellowish centre and orange edge		*Fusarium*	Ameh and Kawo, 2017
SF_6_	Yellow base with white top and yellow taint spreading		*Fusarium*	Ameh and Kawo, 2017
SF_7_	Brown and cotton like		*Penicillium*	Ameh and Kawo, 2017
SF_8_	Black and powdery like		*Aspergillus niger*	Ameh and Kawo, 2017
SF_9_	Deep black and compact			
SF_10_	Like a root system			
SF_11_	Pink			
SF_13_	Pink centre with filamentous on the edges			
SF_14_	Green and powdery light		*Aspergillus flavus*	Ameh and Kawo, 2017
SF_15_	Green centre with white edges			
SF_16_	Black centre with white edges			
SF_17_	Purple centre and white top and edges			
SF_18_	Black and pink edges			
SF_19_	Silver top, brown base with white edges			

**Table 2:** Morphological characteristics of identified bacteria from P greenhouse experiment.

Isolates	Morphology	Special characteristic
SB_1_	Reddish	
SB_2_	Black	
SB_3_	Black centre and white outside	
SB_4_	Pink hallow zoned	Hallow zoned
SB_5_	Cream	
SB_6_	Cream hallow zoned and no other growth nearby	Hallow zoned, inhibitory
SB_7_	Light cream hallow zoned, circle at centre	Hallow zoned
SB_8_	Cream centre and white outside	
SB_9_	Yellow	
SB_10_	Light yellow	
SB_11_	Yellow hallow zoned	Hallow zoned
SB_12_	Yellow and spreading	
SB_13_	Yellow with orange dot at centre	
SB_14_	Yellow centre and cream outside	
SB_15_	Yellow and doom shaped	
SB_16_	Yellow centre and red outside	
SB_17_	Brownish	
SB_18_	Brown at centre with small dot, hallowed zone and light cream spreading	Hallow zoned
SB_19_	Orange	
SB_20_	Whitish with inside typically mitochondria shaped	
SB_21_	White	
SB_22_	White milky spreading	
SB_23_	White doom volcano shaped	
SB_24_	Whitish clear	
SB_25_	Whitish and clearing the media around its growth	Inhibitory
SB_26_	Whitish and tiny	
SB_27_	Whitish hallow zoned	Hallow zoned
SB_28_	Whitish centre and cream radius	
SB_29_	Cream and flowered spreading structure	
SB_30_	Cream with dot at centre	
SB_31_	White, raised edges with centre in a creator	
SB_32_	Whitish spreading flat on the medium	
SB_33_	Cream rod shaped and white growth spreading outward	
SB_34_	Transparent and curled	
SB_35_	Whitish with trellising shape	
SB_36_	Like a flower	
SB_37_	Cream with a creator with flower design	
SB_38_	Transparent centre whitish pale outside	
SB_39_	Whitish balls inside fungi	
SB_40_	Transparent jelly doom shaped	
SB_41_	Transparent spreading	
SB_42_	Tiny circles inside transparent spreading growth	

### Microbial population and dynamics in relation to P rates

**Fig 1:**
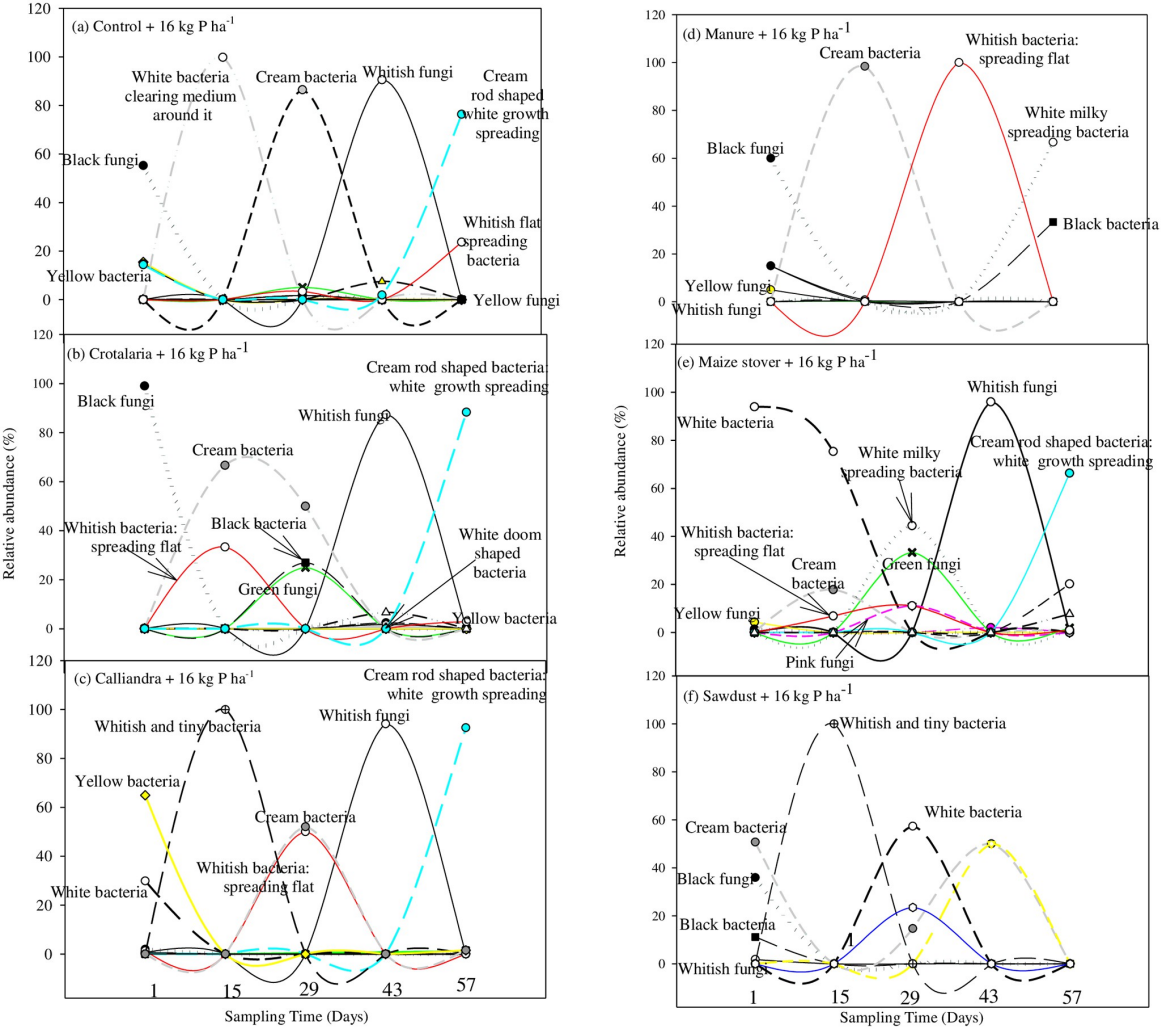
Microbial dynamics following co-application of different quality organics resources and 16 kg P ha^-1^.

On day 1, there was a general dominance of fungi over bacteria except for the 36 kg P ha^-1^ treatment. Legume-based treatments housed > eight (8) species, followed by maize stover which had six (6), while manure, sawdust and the control housed only five (5) species (Fig 1). *Aspergillus niger* had peak abundance (30–98%) across treatments. In the absence or low abundance of *Aspergillus niger*, other microbes proliferated, for example, under *Calliandra*, maize stover and sawdust (Fig 1c, 1e and 1f). Over time, a successional trend characterized by a general microbial diverse peak activity was noted across all treatments on day 29, except under manure (Fig 1). Some microbes were peculiar and suppressed other microbes under the control, *Calliandra* and sawdust on day 15. Regardless of organic resource quality and P rate, *Mucor* and cream rod shaped (*Bacillus*) bacteria peak abundances appeared on day 43 and 57, respectively (Fig 1).

**Fig 2:**
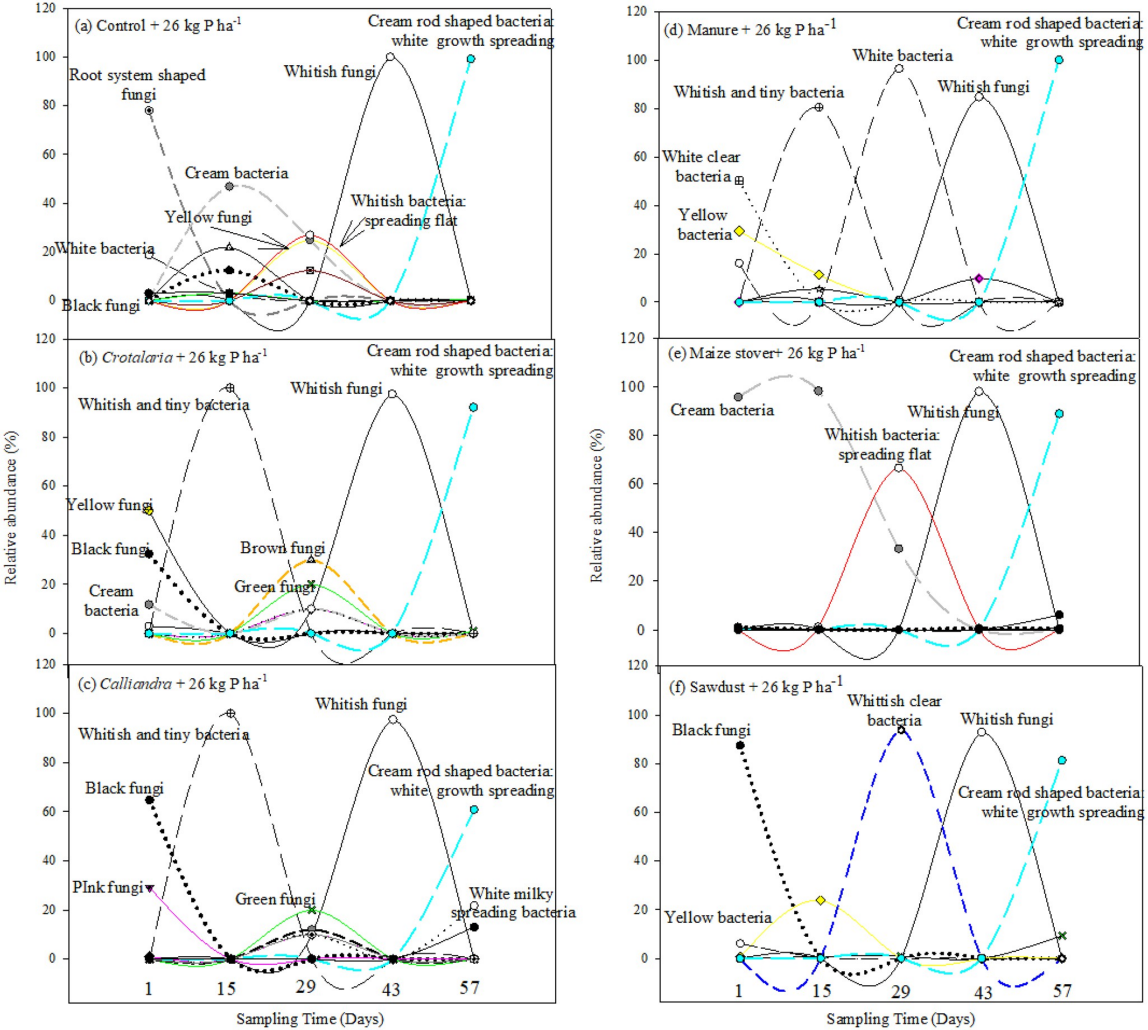
Microbial dynamics following co-application of different quality organics resources and 26 kg P ha^-1^.

Application of 26 kg P ha^-1^ amplified the microbial diverse peak activity on day 29 under the control, *Crotalaria* and *Calliandra*, while whitish spreading flat bacteria dominated under maize stover (Fig 2b–2d). An additional early microbial diverse peak activity was noted under control on day 15 following addition of 26 kg P ha^-1^ (Fig 2a). *Bacillus* emerged under manure and sawdust with the addition of 26 kg P ha^-1^. On day 1 following application of 26 kg ha^-1^, *Aspergillus niger* was stimulated under *Calliandra* and sawdust while its abundance was reduced under *Crotalaria*, control and manure following the application of 26 kg P ha^-1^ (Fig 2). Application of 26 kg P ha^-1^ stimulated white tiny bacteria under *Crotalaria* and manure while the same bacteria was suppressed under sawdust on day 15 (Fig 2).

**Fig 3:**
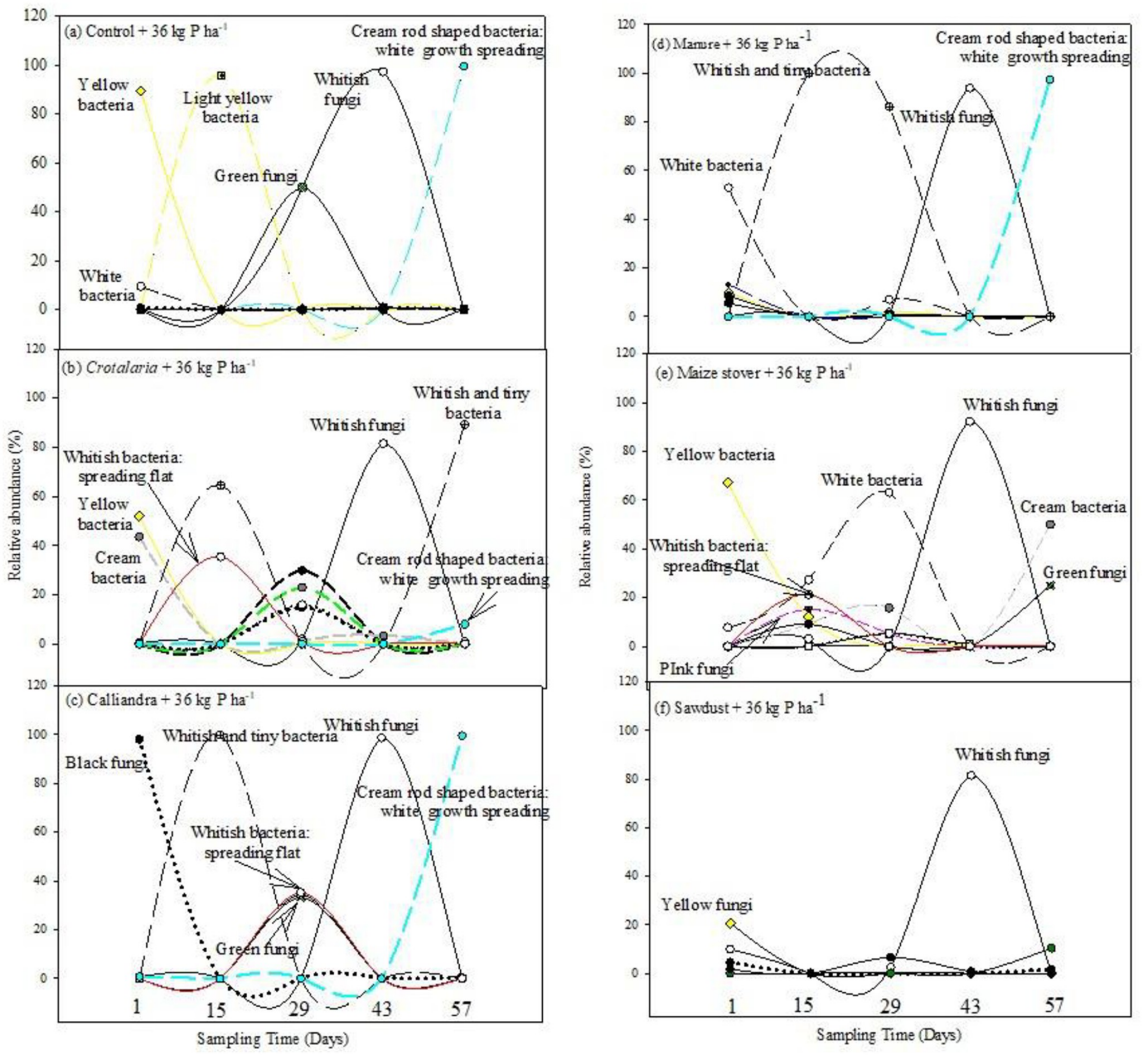
Microbial dynamics following co-application of different quality organics resources and 36 kg P ha^-1^.

Green fungi, yellow and light yellow bacteria were prominent across treatments following the addition of 36 kg P ha^-1^. At the same time, 36 kg P ha^-1^ increased and stimulated *Aspergillus niger* under *Calliandra* to 98% and under sawdust to only 5%, respectively. However, such high P rate suppressed *Bacillus* under maize stover and sawdust (Fig 3e and 3f). Interestingly, the microbial diverse peak activity under control was primarily comprised of fungi (*Mucor* and *Aspergillus falvus*). An additional microbial diverse peak activity was noted under maize stover on day 15 following the addition of 36 kg P ha^-1^ (Fig 3e). Microbes responded differently to increase in P with bacteria being more responsive than fungi. There was a general suppression of cream bacteria across treatments with increase in P. Most bacteria were stimulated at 36 kg P ha^-1^ across the different organic resources. For example, increasing P to 36 kg P ha^-1^ stimulated cream halo-zoned bacteria to 23% under *Crotalaria*, while white bacteria increased to 33% under *Calliandra* and to 63% under maize stover (Figs 1–3).

### Changes in microbial diversity and richness over time

On day 1, the application of sawdust + 36 kg P ha^-1^ resulted in the most diverse microbial population (Hʹ = 1.72) followed by the application of manure + 36 kg P ha^-1^ (Hʹ = 1.48). The lowest diversities were observed under *Crotalaria* + 16 kg P ha^-1^ (Hʹ = 0.08) and *Calliandra* + 36 kg P ha^-1^ (Hʹ = 0.12) (Table 3). Further analysis indicated that the control + 16 kg P ha^-1^ had the maximum species diversity (E = 0.66), while *Crotalaria*, manure with 16 kg P ha^-1^ and *Crotalaria* + 36 kg P ha^-1^ were uneven (E < 0.22). On day 15, the most diverse system was maize stover + 36 kg P ha^-1^ (Hʹ = 1.87) followed by control + 26 kg P ha^-1^ (Hʹ = 1.60) and sawdust + 26 kg P ha^-1^ (Hʹ = 0.99) (Table 3). Over eight treatments were dominated by a single species (Hʹ = 0.0) thus being extremely uneven. *Crotalaria* + 16 kg P ha^-1^ was extremely even (E = 0.94) followed by maize stover + 36 kg P ha^-1^ (E = 0.81). On day 29, the application of *Crotalaria* combined with 26 and 36 kg P ha^-1^ had the highest diversities of Hʹ = 1.83 and Hʹ = 1.88, respectively. Application of manure exhibited the lowest diversities across all P rates (Hʹ = 0.00; Hʹ = 0.20; Hʹ = 0.62) (Table 3). The application of *Calliandra* along with 16 kg P ha^-1^, and maize stover with 26 kg P ha^-1^ resulted in the most maximum species diversities (E > 0.94). Most of the organic resource materials had moderate evenness (E = 0.45–0.95) inclusive of *Crotalaria* and maize stover across all P rates, while five treatments were uneven (E <0.45). On day 43, the treatment that resulted in the most diverse microbial population was *Crotalaria* + 36 kg P ha^-1^ (Hʹ = 0.85) followed by such treatments as *Crotalaria* + 16 kg P ha^-1^, manure + 26 kg P ha^-1^ and sawdust + 36 kg P ha^-1^ (Hʹ = 0.5–0.65). Control + 26 kg P ha^-1^ resulted in the least diverse microbial population (Hʹ = 0.00) (Table 3). On day 57, treatments such as manure + 26 kg P ha^-1^, control + 26 kg P ha^-1^ and control + 36 kg P ha^-1^ resulted in the least microbial population (Hʹ = 0.00–0.06) (Table 3).

**Table 3:** Changes in diversity and evenness across treatments over time.

	Shannon-Wiener (Hʹ)			Evenness (E)		
Time (days)	Lowest	Majority	Highest	Lowest	Majority	Highest
1	Crot at P1(0.075) Call at P3 (0.012)	0.2–1.2	Swd at P3 (1.72) Man at P3 (1.48)	Crot at P1 (0.12) Cal at P3 (0.14)	0.22–0.62	Con, Man at P1; Man at P2; Man at P3 (0.41–0.66)
15	Con, Cal, Swd at P1; Cal, Crot at P2; Man, Swd at P3 (0.00–0.014)	0.1–1.0	Con at P2 (1.60) MzS at P3 (1.87)	Con at P1 (0.15) Swd at P1 (0.13)	0.14–0.81	Crot at P1 (0.95)
29	Man at P1 (0.00)	0.20–1.56	Crot at P2 (1.83) Crot at P3 (1.88)	Man at P2 (0.24) Man at P3 (0.23)	0.51–0.94	Crot, at P1; Con, MzS at P2 (0.94)
43	Con at P2 (0.00)	0.08–0.60	Crot at P3 (0.85)	Crot at P3 (0.19)	.021–0.48	Con, MzS at P1 (0.4–0.48)
57	Con, Swd at P2; Con P3 (0.03–0.67)	0.15–0.85	MzS at P1 (1.05) Cal at P2 (1.04) Crot at P3 (1.04)	MzS at P2 (0.21)	0.23–0.94	Con at P1, Call at P2 (0.70–0.86)

P1 = 16 kg P ha^-1^, P2 = 26 kg P ha^-1^, P3 = 36 kg P ha^-1^, Crot = *Crotalaria*; Cal = *Calliandra*, Man = manure; MzS = maize stover; Swd = Sawdust; Con = control.

### Changes in soil pH, available P and microbial P

**Fig 4:**
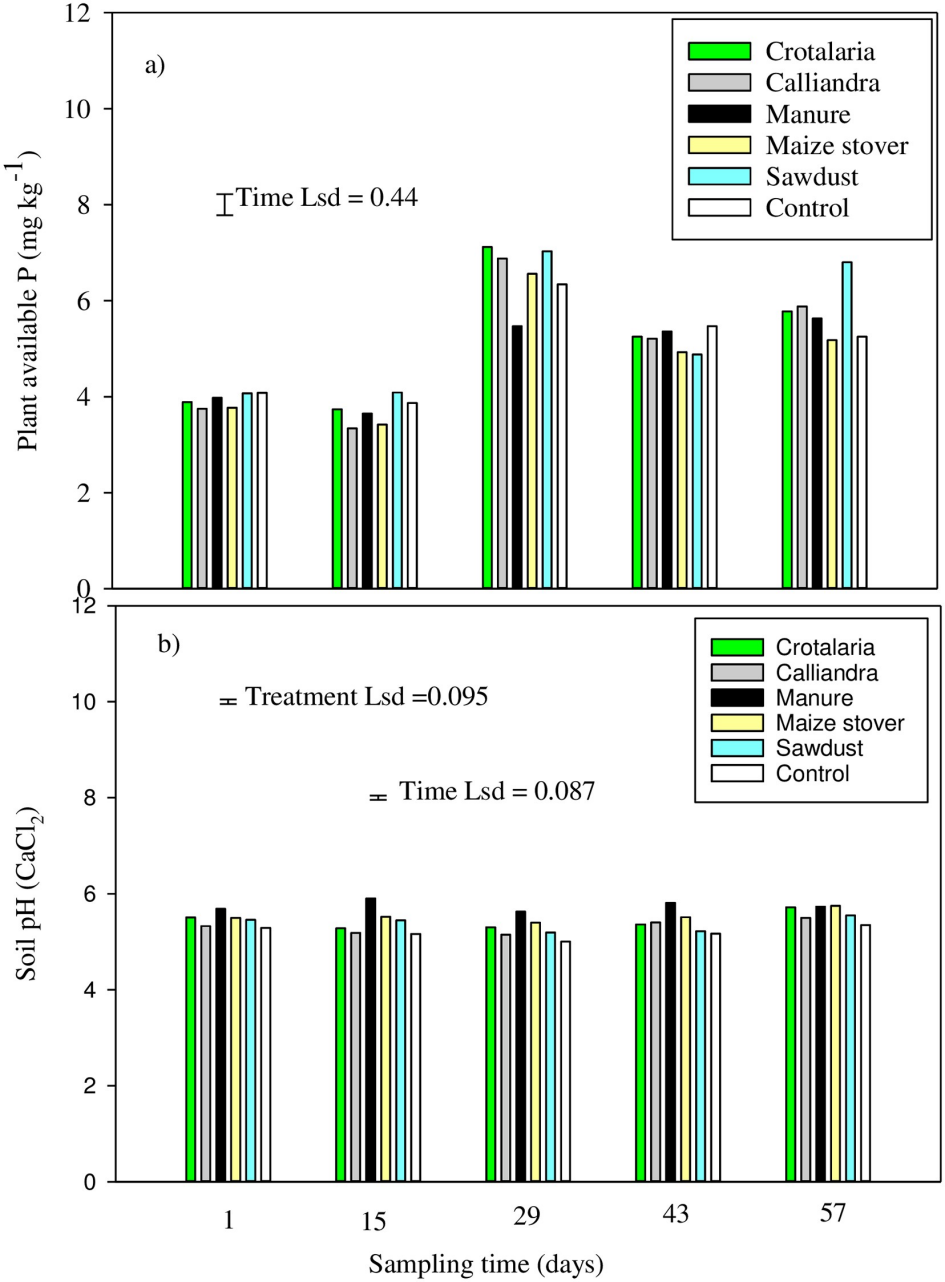
Changes in soil available P and pH across treatments over time.

Despite the difference in organic resource quality, there was no difference in plant available P among treatments at all sampling times (Fig 4a). A significant (P<0.05) rise in available P to between 5.5 and 7 mg kg^-1^ was noted across all organic resources on day 29 (Fig 4). However, the increase in plant available P was not persistent as it declined for all organic resources on day 43. There was a significant (P < 0.05) increase again on day 57 under *Crotalaria*, manure and sawdust (Fig 4a).

Organic resources had a significant (P < 0.05) effect on soil pH on each sampling day. On the first day, the manure treatment had higher soil pH than *Calliandra*, sawdust and the control. Consistently on day 15, the manure treatment had the highest soil pH of 5.9 followed by maize stover and sawdust where soil pH was 5.4 and 5.5, respectively. Consistently, manure recorded significantly higher soil pH than other organic amendments on day 29 and 43 but had similar pH with *Crotalaria* and maize stover at day 57 (Fig 4b). Sampling time had a significant (P< 0.05) effect on soil pH except under sawdust and control, which had almost constant soil pH across all sampling times. Soil pH was not consistent across sampling times under manure and *Calliandra* while *Crotalaria* and maize stover had peak pH on day 57 (Fig 4b).

**Fig 5:**
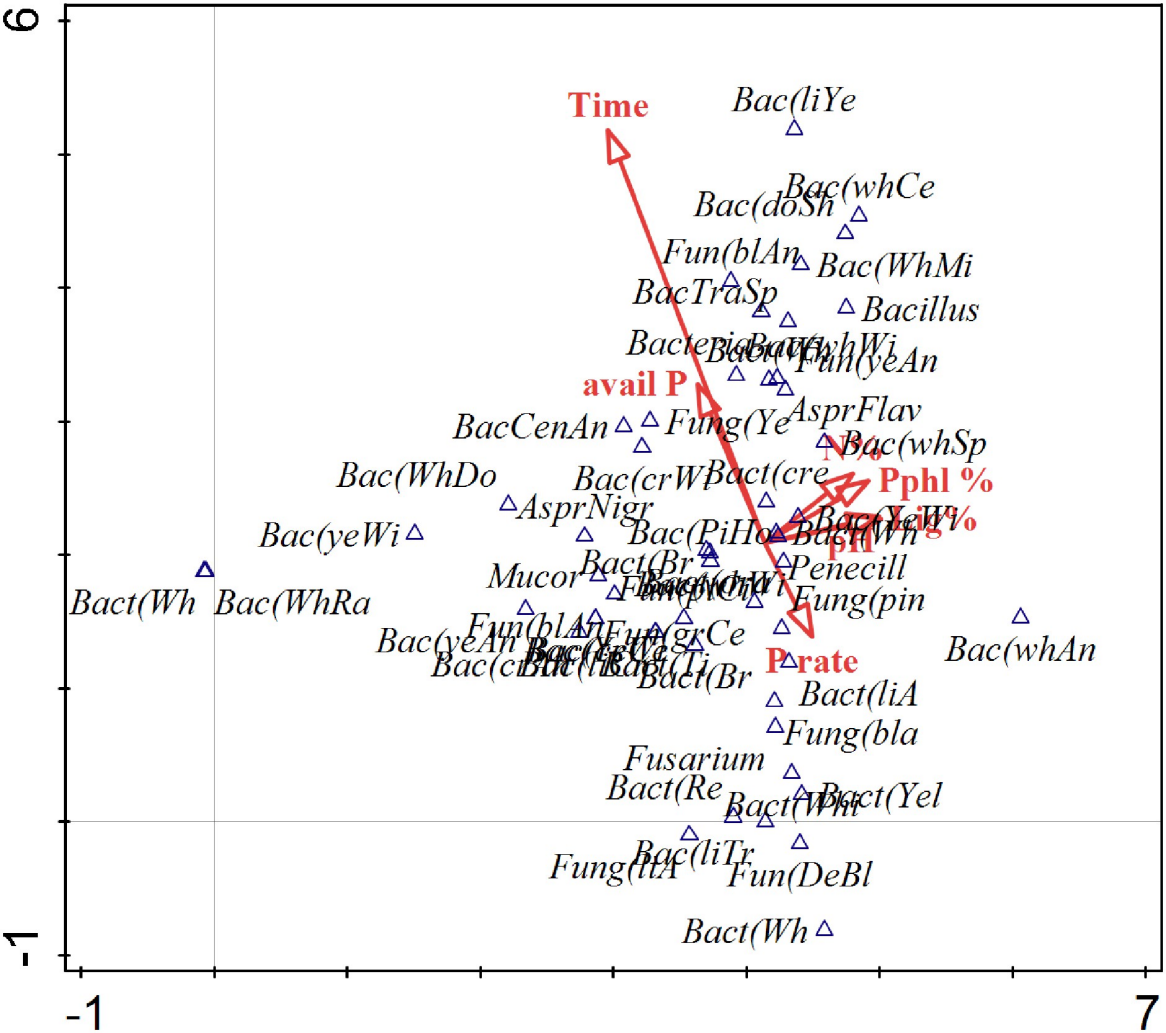
Detrended correspondence analysis (DCA) diagrams of species and environmental variable. The direction of the vectors indicates the direction of maximum change of that variable, whereas the length of the vector represents how well the parameter explains the distribution of the data. External variable (avail P = Plant available phosphorus, Lig% = Lignin content, N% = total N in organic amendment, pH = soil pH, Pphl% = polyphenol content, P rate = P application rate, Time = season time).

Overall, most microbial species were positively correlated to seasonal time and plant available P, while a few species correlated with P rate. Organic resource quality attributes and soil pH had little impact on microbial dynamics. Time was the most influential variable followed by soil available P and P rate (Fig 5). Interactive-forward test indicated that seasonal time and soil available P significantly (P < 0.05) affected microbial community structure (Table 4).

**Table 4:** Detailed results of forward selection test analysis.

External variables	Explains %	Contribution %	pseudo-F	P
**Time**	5.1	39.1	4.7	0.002
**Avail P**	2.5	19.4	2.4	0.002
**Lig%**	1.3	9.7	1.2	0.2
**N%**	1.2	9.0	1.1	0.298
**P rate**	1.2	9.0	1.1	0.304
**pH**	1.1	8.1	1.0	0.462
**Pphl %**	0.7	5.7	0.7	0.894

Avail P = Plant available phosphorus, Lig% = Lignin content, N% = total N in organic amendment, pH = soil pH, Pphl% = polyphenol content, P rate = P application rate, Time = season time

Both organic resources and P rate had a significant (P <0.01) effect on microbial P over the sampling time points (day 1 to day 57). However, sampling time had no effect on microbial P. Following the application of 16 kg P ha^-1^ on day 1, microbial P was highest under *Crotalaria* and maize stover while the least was under *Calliandra* (Table 5). On day 1, the *Crotalaria* + 26 kg P ha^-1^ treatment recorded in the highest microbial P compared with the other organic resources and the control. At 36 kg P ha^-1^, the control and manure had the highest microbial P than *Calliandra* > maize stover > *Crotalaria* (Table 5). Increasing P from 16 to 26 kg P ha^-1^ significantly increased microbial P under *Crotalaria*, *Calliandra* and sawdust. Further increase to 36 kg P ha^-1^ increased P immobilization under *Calliandra* to 87 mg/kg, while microbial P was reduced to < 30 mg/kg under *Crotalaria* and sawdust (Table 5).

**Table 5:** Changes in soil microbial P over time following co-application of organic resource with varying P rates.

Time (days)		Soil microbial P (mg kg^-1^ soil)			
	Treatments	16 kg P ha^-1^	26 kg P ha^-1^	36 kg P ha^-1^	*Lsd*
1	*Crotalaria*	190^a,i^	277^a,ii^	20^d,iii^	***11***
	*Calliandra*	40^c,i^	63^d,ii^	87^b,iii^	***11***
	Manure	160^b,i^	-113^f^	127^a,ii^	***11***
	Maize stover	220^a,i^	103^c,ii^	63^c,iii^	***11***
	Sawdust	123^b,i^	153^b,ii^	-30^e,iii^	***11***
	Control	130^b,i^	13^e,ii^	127^a,i^	***11***
	*Lsd*	***16***	***16***	***16***	
15	*Crotalaria*	-110^d,i^	157^bc,ii^	67^c,iii^	***14***
	*Calliandra*	230^a,i^	237^a,i^	170^a,ii^	***14***
	Manure	93^b,i^	110^d,i^	120^b,ii^	***14***
	Maize stover	20^c,i^	233^a,ii^	3^d,i^	***14***
	Sawdust	117^b,i^	123^cd,i^	-67^e,ii^	***14***
	Control	157^b,i^	167^b,i^	77^c,ii^	***14***
	*Lsd*	***20***	***20***	***20***	
29	*Crotalaria*	147^b,i^	170^b,ii^	-10^iii^	***8***
	*Calliandra*	163^b,i^	207^a,i^	-83^iii^	***8***
	Manure	83^d,i^	220^a,ii^	-70^iii^	***8***
	Maize stover	77^d,i^	213^a,ii^	0^iii^	***8***
	Sawdust	120^c,i^	20^d,ii^	50^a,iii^	***8***
	Control	333^a,i^	70^c,ii^	-27^iii^	***8***
	*Lsd*	***11***	***11***	***11***	
43	*Crotalaria*	-53	-14	53^d^	***12***
	*Calliandra*	240^a,i^	77^c,ii^	0^e,iii^	***12***
	Manure	127^d,i^	110^b,i^	123^c,i^	***12***
	Maize stover	150^c,i^	287^a,ii^	230^a,iii^	***12***
	Sawdust	180^bc,i^	71^c,ii^	150^b,iii^	***12***
	Control	200^b,i^	100^bc,ii^	40^d,iii^	***12***
	*Lsd*	***16***	***16***	***16***	
57	*Crotalaria*	340^a,i^	130^a,ii^	230^a,iii^	***13***
	*Calliandra*	40^cd,i^	28^cd,i^	-53	***13***
	Manure	70^c,i^	27^cd,ii^	-23	***13***
	Maize stover	137^b,i^	13^d,ii^	10^d,ii^	***13***
	Sawdust	30^d,i^	50^bc,ii^	117^b,iii^	***13***
	Control	73^c,i^	80^b,i^	63^c,i^	***13***
	*Lsd*	***19***	***19***	***19***	

*Means followed by the same letters and roman number superscript in columns and ***rows*** respectively are no significantly different at P<0.05

Fifteen days after application of 16 kg P ha^-1^ the microbes under *Calliandra* and *Crotalaria* immobilized the highest and least P, respectively (Table 5). At 26 kg P ha^-1^, microbial P was highest under *Calliandra* and maize stover > *Crotalaria* = control > sawdust > manure. At 36 kg P ha^-1^, *Calliandra* had the highest microbial P than manure > *Crotalaria =* control *>* maize stover on day 15, while no immobilization was recorded under sawdust (Table 5). Increasing P from 16 to 26 kg P ha^-1^ significantly reduced microbial P under *Crotalaria*, while no changes in microbial P were noted under *Calliandra*, sawdust and control. Increasing P to 36 kg ha^-1^ decreased immobilization under *Crotalaria*, *Calliandra*, sawdust and control, while microbial P under manure remained constant. On day 29, microbial P was highest under control > *Crotalaria* = *Calliandra* > sawdust > manure following application of 16 kg P ha^-1^ (Table 5). Addition of 26 kg P ha^-1^ gave highest microbial P under manure, maize stover and *Calliandra* followed by *Crotalaria* > control > sawdust. Increasing P to 26 kg ha^-1^ under *Crotalaria*, *Calliandra*, manure and maize stover significantly increased microbial P, while it reduced under sawdust and control. However, further addition of P to 36 kg ha^-1^ significantly reduced microbial P across treatments except under sawdust (Table 5).

On day 43 under 16 kg P ha^-1^, microbial P was highest under *Calliandra* > sawdust = control > maize stover > manure (Table 5). At 26 kg P ha^-1^, microbial P was highest under maize stover followed by manure = control > *Calliandra* = sawdust. Consistently, maize stover attained the highest microbial P following addition of 36 kg P ha^-1^ followed by sawdust and manure (Table 5). Increasing P under *Crotalaria*, *Calliandra* and control significantly reduced microbial P on day 43, while no change was noted under manure (Table 5). On day 57, microbial P was highest under *Crotalaria* > maize stover > *Calliandra* = manure = control at 16 kg P ha^-1^ (Table 5). At 26 kg P ha^-1^, microbial P was highest under *Crotalaria* followed by sawdust. Consistently at 36 kg P ha^-1^, *Crotalaria* attained the highest microbial P followed by sawdust (Table 5).

### The effect of increasing P on maize growth attributes and P uptake

**Fig 6:**
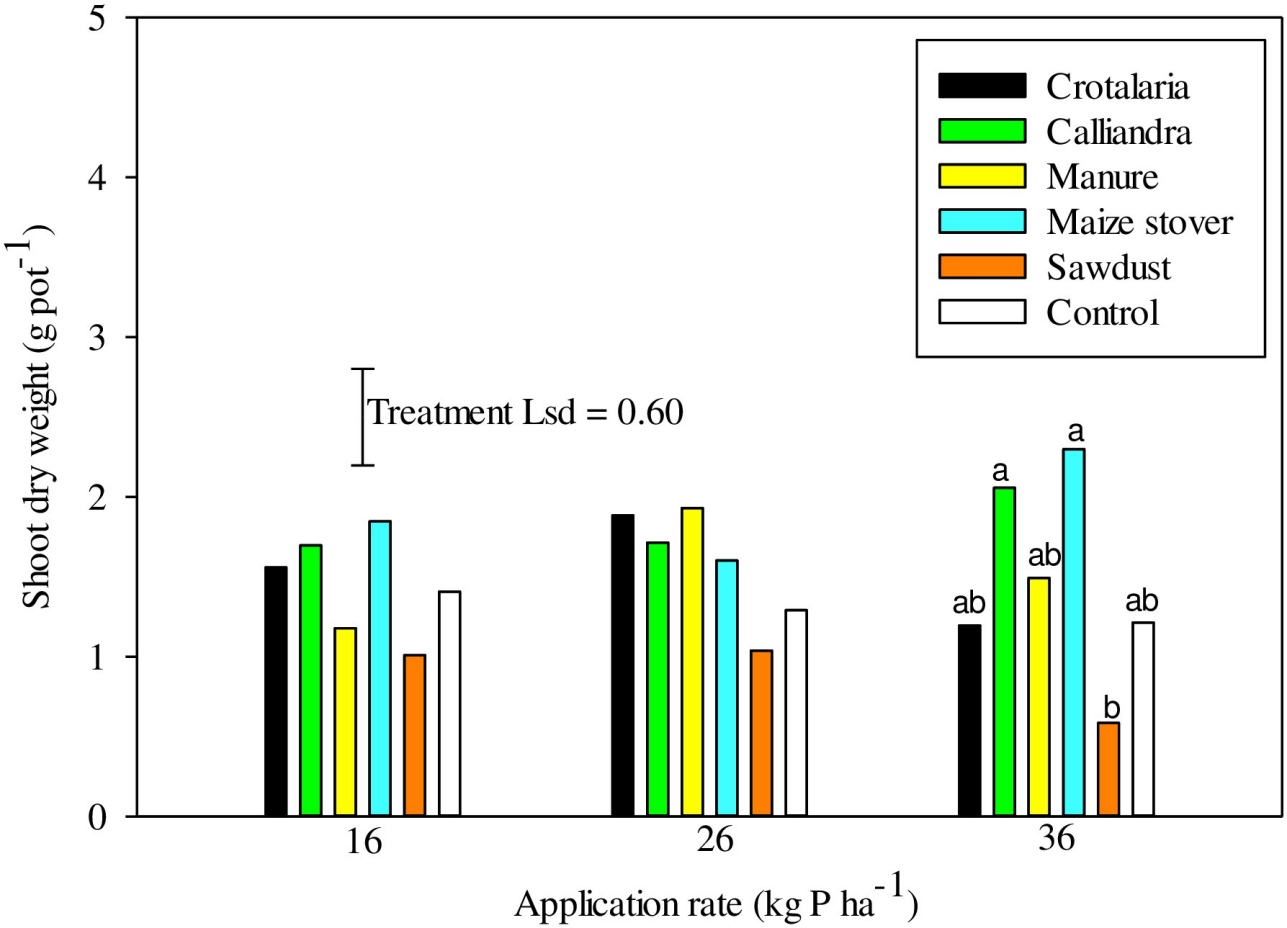
Effects of treatment on shoot dry weight at harvesting across three P rates.

**Fig 7:**
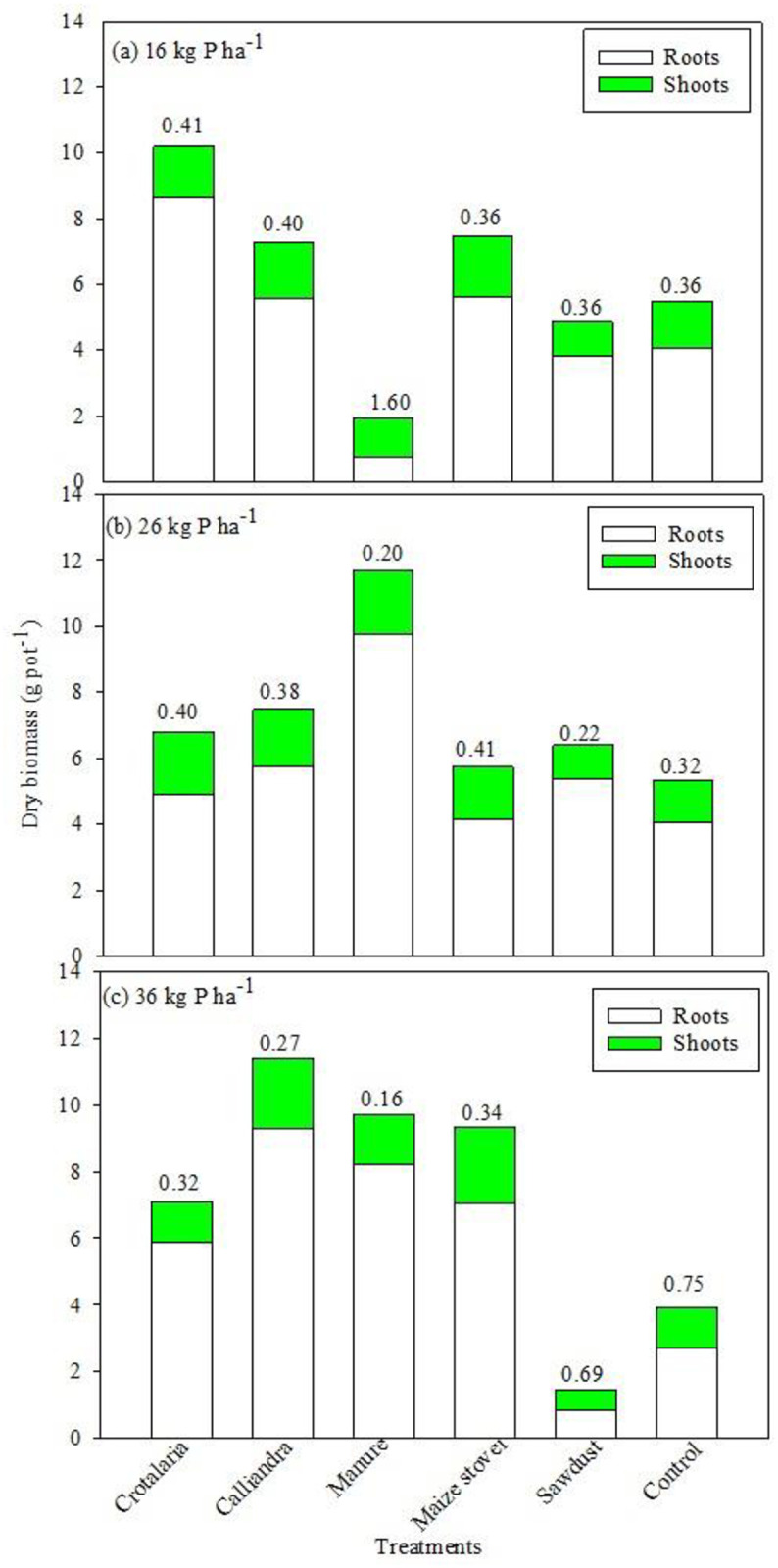
Biomass productivity and shoot to root ration at harvesting following P and P+N application on treatments. Figures on top of the bars are S: R for the treatments.

Increasing the P rate from 16 to 36 kg P ha^-1^ had no effect on maize shoot dry weight (SDW) across all organic resources. Significant (P < 0.05) differences in SDW were observed solely at 36 kg P ha^-1^ under *Calliandra* and maize stover (Fig 6). Unexpectedly, there was no organic resources and P rate effects on root dry weight. However, application of P had a significant (P<0.05) effect on shoot to root ratio (S: R) (Fig 7a–7c). There was a significant (P< 0.01) organic resource and P interaction on S: R. Increasing P beyond 26 kg ha^-1^ under manure significantly (P<0.05) reduced S: R from 1.6 to < 0.2. On the contrary, increasing P rate to 36 kg ha^-1^ significantly (P<0.05) increased S: R to 0.69 and 0.75 under sawdust and control, respectively (Fig 7a–7c). However, there was no organic resource and P rate effect on shoot, root and total P uptake following incremental sole supply of P across treatments.

## Discussion

### Microbial dynamics following co-application of organic and inorganic resources

Microbes identified across treatments were P-solubilizing given that the PVK media supports the proliferation of such microbes. *Bacillus*, *Mucor*, *Fusarium*, *Penicillium* and two *Aspergillus* species have previously been identified as key P-solubilizing fungi [52, 58, 59]. The presence of hallow and solubilizing zone for some bacteria is confirmation of their P solubilizing capacity. Proliferation of *Mucor* and *Bacillus* at high soil available P is suggestive of their involvement in P solubilization or a high demand for P. The isolation and identification of *Fusarium*, *Penicillium*, *Aspergillus* and *Bacillus* species confirms metagenomics results [34], supporting the need to combine both dynamic and static approaches in soil microbial studies. Further studies could explore metagenomics analysis of microbial colony samples to authenticate species identified in this study. The plate-counting technique used in this study is a dynamic approach capable of detecting both active and potentially active microbes that are directly linked to nutrient cycling processes [45]. As such, there is room for the development of effective fungi or bacteria based biofertilizers to help alleviate soil P deficiency in most smallholder farming systems in Zimbabwe and the region in the wake of projected rise in prices of P based fertilizers [17, 22, 23].

The hyphal and filamentous growth in fungi support better water and nutrients utilization in dry soils that would otherwise be unavailable to bacteria [60, 61] which explain high fungal dominance on day 1 compared to bacteria which are usually dormant in the initial stages of soil wetting. As such fungi would multiply at the expense of the dormant bacteria. On the other hand, dry soil conditions can stimulate some fungi (e.g., *Penicillium* and *Aspergillus* species) to produce inactive spores [61] that germinate fast when environmental conditions improve. In this case, the addition of both water and P in the soil [62] stimulated germination of various spores and general microbial activity [63]. Detection of microbes on day 1 also indicate the presence of potentially active microbes that are in a physiological alertness to use any applied suitable resource [45, 64, 65]. Such processes typify the onset of the rainfall season as trigger molecules, leached nutrients and soluble carbon are released due to the sudden change in soil moisture from dry soils, similar to the typical Birch effect reported in previous studies [15, 63, 64, 66, 67]. Moreover, the fungi dominance at the onset, and succession of fungi and bacteria over the study period is typical during decomposition [34, 61]. More species taxa on day 1 under legume-based organic nutrient resources indicate higher concentration of trigger molecules and soluble substrates from the decomposing organics than under low-quality organic nutrient resources [34]. The same reasons could explain the high diversities between days 29 and 57 under combinations of medium to high quality organic resources and 26 or 36 kg P ha^-1^ [33, 34, 70]. Microbial proliferation is due to environmental conditions that ensure ready supply of food or energy and less of the toxins or detrimental factors [34, 68, 69]. Apart from the general requirements, other microbes have special nutritional or environmental needs that further improve their proliferation and effectiveness. This study concurs with several studies on the concept of microbial preferences for certain resources to proliferate [33, 34, 70] as indicated by nutrient resource quality related preference and peculiarity.

The coinciding of diversity increase, microbial diverse peak activity and high plant available P on day 29 indicate a P starvation period for the planted crop. These results support the outcome of multivariate analysis that indicated importance of seasonal time and available P in manipulating microbial community structure as decomposition proceeds. Since there were no changes in microbial P over time, the increase in plant available P can be partly linked to solubilization of insoluble phosphate P rather than microbial turnover. This study mainly focused on P solubilizing microbes (fungi and bacteria), which make it difficult to relate to microbial P, a product of lysis of total soil microbes. At the same time, such high microbial P indicates that most of the soil P was immobilized, implying high P demand by microbes. Stimulation or increase in relative abundance of microbes such as *Aspergillus niger* (under *Crotalaria* and sawdust), yellow and light yellow bacteria (under control, maize stover and *Crotalaria*) following application of 36 kg P ha^-1^ confirms the high P demand by microbes. This build up of microbial P can be linked to the soil organic carbon stabilization at high application rate highlighted by Mtangadura et al. [44]. Despite such high microbial P and high diversity, functionality of microbes is key for the solubilization of insoluble P for the crop.

The low available P in the first two weeks of the incubation study suggests that most of the fertilizer P and that from decomposition of SOM were immobilized by soil microbes. The immobilization can potentially reduce P leaching losses [71]. During the early immobilization period, the crop would not have developed a good rooting system to harness the P. As was shown in this study, available P increased after day 29 onwards thereby presenting better synchrony between nutrient release and crop demand. These results point to the need for farmers to plant their crops within one month of co-application of organic and inorganic nutrient resources to synchronize the rise in available P and utilization by crops versus potential leaching loss. In China, various technologies are being employed to increase the C: P of organic materials to promote P immobilization, enhance synchronization between nutrient and plant demand, and subsequently reduce environmental damage [32]. The slight microbial diverse peak activity under the control indicates the importance of organic resources application to supply food or energy for microbes and macrofauna to multiply [33, 34]. At the same time, the early microbial diverse peak activity under the control on day 15, and selective stimulations and amplification of microbial diverse peak activity on day 29 under *Crotalaria* and *Calliandra* following application of 26 kg P ha^-1^ indicate that P was limiting the system at 16 kg P ha^-1^, and therefore the need to increase P application rate. Such responses from *Crotalaria* and *Calliandra* show that their P content was low to support microbial activities and thus mineral P basal fertilizers should also be added to support both soil microbial activity and crop growth. Selective species (e.g., Brown fungi under *Crotalaria*) which had low abundances or absent responded to the increase in P while others that dominated at 16 kg ha^-1^ were suppressed (e.g., cream bacteria under control, *Crotalaria*, *Calliandra* and manure) causing the amplified microbial diverse peak activity on day 29. This shows that microbes (within PS fungi/bacteria species or genus) differ in their P requirements and utilization efficiencies in soils as affected by the organic resource quality applied. The positive response of microbes to P under manure indicates that manure alone is a poor source of P confirming findings by Wuta and Nyamugafata [16]. This concurs with the fact that most organic resources in smallholder farming systems have low P content to support maize growth [39, 72, 73]. Therefore, addition of P based fertilizer at planting is a must to reduce P deficiency to both crops and microbes.

Differences in P response by bacteria/fungi is typical as microbial species differ in their ability, efficiency and mechanism to acquire or solubilize the resource [74–77]. Microbes have specific P requirement (low or high demand) while very high P rates may reduce relative abundance but linked to resource quality. The suppression of *Bacillus* under maize stover and sawdust following addition of 36 kg P ha^-1^ indicates that other nutrients than P were now limiting its growth typical of the Sprengel-Liebig Law of the Minimum [77]. The maintained trajectories for *Mucor*, white tiny and *Bacillus* under *Crotalaria*, *Calliandra*, manure and control at 36 kg P ha^-1^ suggest that 26 kg P ha^-1^ was enough to meet their requirements. An early microbial diverse peak activity on day 15 under maize stover following addition of 36 kg P ha^-1^ points to the adequate supply of P for microbial activation and diversity under such a low-quality resource. On the other hand, the concept of diminishing returns could have also affected some microbes (e.g., *Mucor*, *Fusarium*, *Aspergillus flavus* and *Bacillus*) in specific organic resources. Microbes require P to sustain their survival as indicated by the high microbial P, but under P limiting conditions microbes will initiate P solubilization process to meet their need. However, P solubilization is an energy demanding process for microbes, similar to biological nitrogen fixation [78], and under high soil available, P solubilization processes are suppressed or deactivated. As such, cereal-based organic nutrient resources, with high C: P, should be co-applied with high rates of fertilizer P to stimulate microbial diversity that promotes decomposition and nutrient cycling. Similarly, most low quality organic nutrient resources, such as sawdust, are also typified by high C: N and would therefore would require fertilizer N to offset immobilization [79–81].

### The effect P on maize growth and P recovery

Increasing P alone without supplying complementary nutrients did not benefit maize shoot and root dry matter accumulation. In addition poor sources of most macro-and micronutrients, most medium and low quality resources have high C: P and C: N ratios which promote immobilization and limiting available N and P for plant growth. Similarly, several studies have highlighted high P uptake and maize yields following addition of N and other macro-and micronutrients to the soil [24, 82–85]. Phosphorus is known to be critical in root development and establishment [86] particularly for legumes that can make their own N from biological fixation. However, cereals cannot make their own nitrogen; as such fertilizers or soils must supply both N and P, and other nutrients to support crop development [82, 87–89]. Basing on this study, farmers need to add high rates of mineral P based fertilizers to stimulate soil microbial activity and offset immobilized P in microbes. Sole application of P and increasing P rate to 36 kg P ha^-1^ to different quality organic resources had no effect on shoot, root and total P uptake as the system was limited by other nutrients such as nitrogen [90, 91]. Tekulu et al. [92] reported that balanced fertilization promotes cell division and meristematic development in plants. Increasing P across treatments ameliorated P deficiency but other nutrients e.g. N could have become limiting [77]. Crops are able to detect soil nutrients levels and send signals within the plant that ensure survival of the crop particularly on distribution of potential recoverable nutrients detected [93]. As such, application of medium to high quality nutrient resources in farmers’ fields will benefit both the root and shoot systems of the maize crop [90, 91].

Overall, a limitation of our study is that it was conducted over a short period and under a controlled environment. For example, soil moisture content was controlled over the experimental period, which is somewhat different to rain-fed field conditions where soil water dynamics vary as detected by rainfall events and farmer management practices. The low maize P uptake and recovery reported in study could have been because measurements were only done up to 57^th^ day after planting. Results could have been different if measurement were conducted up to physiological maturity of the maize crop. A previous study showed that P uptake normally continues linearly in shoot component of maize up to physiological maturity [94]. There is therefore need for long-term field studies to argument our findings. Regarding characterization of soil microbial dynamics, further studies could explore DNA analysis of microbial colony samples to authenticate species identified in this study.

## Conclusions

This study showed that soil bacteria are more responsive to applied P than fungi. Microbial phosphorus requirement is dependent on species with some requiring less and others having high demand. Successional trend was apparent across treatments at different P rates (16, 26 and 36 kg P ha^-1^) with trajectory of some microbes (*Mucor*, white tiny bacteria and *Bacillus*) maintained, coupled with a microbial diverse peak activity on day 29. Increasing P application rate amplified microbial diverse peak activity on day 29 or triggered an early bulge on day 15 depending on organic resource quality. Interactive-forward test indicated that seasonal time and soil available P were most influential (P < 0.05) factors shaping microbial communities. Farmers need to add high rates of mineral P based fertilizers to stimulate soil microbial diverse peak activity and offset microbial immobilized P. However, increasing P rate alone has no effect on maize growth and P uptake, as other nutrients might become limiting to complement the P increase. This study showed adding medium-high quality organic resources coupled with high P rate (>26 kg P ha^-1^), is key in the development of a balanced fertilization strategy to stimulate soil microbial growth and diversity, increase soil available P and increase maize growth on sandy soils. Our results suggest the need to reconsider current P fertilizer recommendations for maize production on sandy soils as well develop new fertilizer formulations to intensify maize cropping in Zimbabwe.

## Supporting information

S1 Table Initial soil properties of the top 20 cm at Domboshawa, Zimbabwe. (DOC)

S2 Table Quality attributes of the organic resources used in experiment. (DOC)
