# Supplementary material for: Increasing phosphorus rate alters microbial dynamics and soil available P in a Lixisol of Zimbabwe
Source: PLoS One. 2023 Sep 8;18(9):e0291226. doi: 10.1371/journal.pone.0291226 (PMC10490935; doi:10.1371/journal.pone.0291226)
Supplement: S1 Table — (DOC) [file pone.0291226.s001.doc]

Table S1. Initial soil properties of the top 20 cm at Domboshawa, Zimbabwe.

| Clay% | Silt% | Sand% | Organic C (%) | Total N (%) | Available P (ppm) | pH (CaCl_2_) | Exchangeable cations  (cmol kg^-1^) | | |
| --- | --- | --- | --- | --- | --- | --- | --- | --- | --- |
|  |  |  |  |  |  |  | Ca | Mg | K |
| 22 | 5 | 73 | 0.73  (1) | 0.07  (0.1) | 6.3  (0.51) | 4.5  (0.3) | 0.8 (0.1) | 0.7 (0.06) | 0.2 (0.04) |

Figures in parentheses indicate standard errors (Adapted and modified from Mtambanengwe et al., 2006)
