# Supplementary material for: Increasing phosphorus rate alters microbial dynamics and soil available P in a Lixisol of Zimbabwe
Source: PLoS One. 2023 Sep 8;18(9):e0291226. doi: 10.1371/journal.pone.0291226 (PMC10490935; doi:10.1371/journal.pone.0291226)
Supplement: S2 Table — (DOC) [file pone.0291226.s002.doc]

Table S2. Quality attributes of the organic resources used in experiment

| **Quality parameters** | **Organic resource** | | | | |
| --- | --- | --- | --- | --- | --- |
|  | ***Crotalaria juncea*** | ***Calliandra calothyrsus*** | **Cattle manure** | **Maize stover** | ***Pinus patula* sawdust** |
| Carbon (g kg^-1^) | 45 | 45 | 31 | 45 | 44 |
| Nitrogen (g kg^-1^) | 44 | 32 | 9 | 6 | 0.4 |
| Lignin (g kg^-1^) | 32 | 115 | 83 | 11 | 295 |
| Polyphenols (g kg^-1^) | 30 | 121 | 2 | 295 | 17 |
| C/N ratio | 10 | 14 | 31 | 69 | 122 |
| Overall quality status | High | Medium | Variable | Low | Very low |

Adopted and modified from Mtangadura et al., 2017
